# Polymorphisms in the estrogen receptor alpha gene (*ESR1*), daily cycling estrogen and mammographic density phenotypes.

**DOI:** 10.1186/s12885-016-2804-1

**Published:** 2016-10-07

**Authors:** F. N. Fjeldheim, H. Frydenberg, V. G. Flote, A. McTiernan, A-S Furberg, P. T. Ellison, E. S. Barrett, T. Wilsgaard, G. Jasienska, G. Ursin, E. A. Wist, I. Thune

**Affiliations:** 1 The Cancer Centre, Oslo University Hospital, Oslo, N-0424 Norway; 2 Institute of Clinical Medicine, University of Oslo, Oslo, N-0316 Norway; 3 Fred Hutchinson Cancer Research Center, Public Health Sciences Division, Seattle, WA USA; 4 Department of Community Medicine, Faculty of Health Sciences, UiT The Arctic University of Norway, 9037 Tromsø, Norway; 5 Department of Microbiology and Infection Control, University Hospital of North Norway, 9038 Tromsø, Norway; 6 Department of Anthropology, Harvard University, 11 Divinity Avenue, Cambridge, MA 02138 USA; 7 Department of Obstetrics and Gynecology, University of Rochester School of Medicine and Dentistry, Rochester, NY 14642 USA; 8 Department of Environmental Health, Institute of Public Health, Jagiellonian University Medical College, Grzegorzecka 20, Krakow, 31-351 Poland; 9 Cancer Registry of Norway, PO Box 5313, Majorstuen Oslo, N-0304 Norway; 10 Department of Clinical Medicine, Faculty of Health Sciences, UiT The Arctic University of Norway, 9037 Tromsø, Norway

**Keywords:** Polymorphisms, Mammographic density, ESR1, 17β-estradiol, Premenopausal

## Abstract

**Background:**

Single nucleotide polymorphisms (SNPs) involved in the estrogen pathway and SNPs in the estrogen receptor alpha gene (ESR1 *6q25*) have been linked to breast cancer development, and mammographic density is an established breast cancer risk factor. Whether there is an association between daily estradiol levels, SNPs in *ESR1* and premenopausal mammographic density phenotypes is unknown.

**Methods:**

We assessed estradiol in daily saliva samples throughout an entire menstrual cycle in 202 healthy premenopausal women in the Norwegian Energy Balance and Breast Cancer Aspects I study. DNA was genotyped using the Illumina Golden Gate platform. Mammograms were taken between days 7 and 12 of the menstrual cycle, and digitized mammographic density was assessed using a computer-assisted method (Madena). Multivariable regression models were used to study the association between SNPs in *ESR1*, premenopausal mammographic density phenotypes and daily cycling estradiol.

**Results:**

We observed inverse linear associations between the minor alleles of eight measured SNPs (*rs3020364*, *rs2474148*, *rs12154178*, *rs2347867*, *rs6927072*, *rs2982712*, *rs3020407*, *rs9322335*) and percent mammographic density (*p*-values: 0.002–0.026), these associations were strongest in lean women (BMI, ≤23.6 kg/m^2.^). The odds of above-median percent mammographic density (>28.5 %) among women with major homozygous genotypes were 3–6 times higher than those of women with minor homozygous genotypes in seven SNPs. Women with *rs3020364* major homozygous genotype had an OR of 6.46 for above-median percent mammographic density (OR: 6.46; 95 % Confidence Interval 1.61, 25.94) when compared to women with the minor homozygous genotype. These associations were not observed in relation to absolute mammographic density. No associations between SNPs and daily cycling estradiol were observed. However, we suggest, based on results of borderline significance (*p* values: 0.025–0.079) that the level of 17β-estradiol for women with the minor genotype for *rs3020364*, *rs24744148* and *rs2982712* were lower throughout the cycle in women with low (<28.5 %) percent mammographic density and higher in women with high (>28.5 %) percent mammographic density, when compared to women with the major genotype.

**Conclusion:**

Our results support an association between eight selected SNPs in the *ESR1* gene and percent mammographic density. The results need to be confirmed in larger studies.

**Electronic supplementary material:**

The online version of this article (doi:10.1186/s12885-016-2804-1) contains supplementary material, which is available to authorized users.

## Background

Genetic factors are believed to account for 30–60 % of the variance in mammographic density [1, 2] while established breast cancer risk factors such as age, body mass index (BMI), parity, age at first birth, the use of hormone therapy and physical activity account for the remainder of its variability [3]. Substantial evidence supports the role of ovarian steroid hormones in breast cancer [4], and estrogens are associated with breast cancer development in both pre- and postmenopausal women [5–7]. Estrogens increase cellular proliferation in breast tissue, which may increase mammographic density [8]. Mammographic density reflects the proportion of fibroglandular cells in the breast tissue; higher density indicates increased potential for proliferative activity [9] and is an established risk factor for breast cancer [10, 11]. Recently, studies of associations between breast cancer risk factors and mammographic density has focused not only on percent mammographic density [12, 13], but on various mammographic density phenotypes [14–17] as absolute mammographic density is believed to represent the actual target tissue for tumor development [18–21].

The genetic determinants of mammographic density have yet to be identified. A recent combined meta analysis of data from five genome wide studies (GWAS) among women of European decent suggests that multiple loci might be involved [22, 23]. Given the associations between estrogens and breast cancer, it is plausible that genetic variation in estrogen receptors may be important.

Both estrogen receptors, alpha (ERα) and beta (ERβ), are members of the nuclear receptor family of ligand-inducible transcription factors, but they are believed to have different transcriptional activation properties [8]. It has been assumed for a long time that the action of estrogens in carcinogenesis is via the ERα signaling and the proliferation induced by the interaction between estrogens and estrogen receptors. [4, 24] Thus, the estrogen receptor alpha gene (*ESR1*), which encodes ERα, is of special interest in relation to breast cancer initiation, development, and therapeutics. Specific polymorphisms (SNPs) in *ESR1* may directly or indirectly lead to variations in its activity, and may have an effect on breast cancer risk. Although a number of studies have examined SNPs located in estrogen related genes and possible associations with mammographic density [25–36], few have focused on premenopausal women [25, 27, 28, 33, 36], and only a handful have considered SNPs in the *ESR1* (*rs2234693* and *rs9340799*) [25, 26, 29–31]. Further, none have chacterized participants by menstrual cycle hormonal milieu.

Previously, in the Norwegian Energy Balance and Breast Cancer Aspects (EBBA)-I study, we observed a positive association between daily circulating ovarian sex hormones and both absolute and percent mammographic density [15, 37]. In addition, daily 17β-estradiol profiles were positively associated with traditional breast cancer risk factors, such as early age at menarche [38], short time since last birth [39], and unfavourable metabolic profile [38, 40, 41]. We also observed associations between two SNPs in the estrogen pathway (rs7172156 and rs749292 in CYP19A1), daily cycling 17β-estradiol, and absolute and percent mammographic density [17] as well as between CYP17 (*rs2486758*) and metabolic risk factors [42] These associations also point to the need for further studies of estrogen, mammographic density phenotypes and susceptibility genes in combination. Here, we expand upon those results to consider the ESR1 region, examining whether SNPs are associated with daily cycling estradiol and premenopausal mammographic density phenotypes.

## Methods

### Participants and study design

The Norwegian Energy Balance and Breast cancer Aspects I study (EBBA-I) included a total of 204 healthy premenopausal women, aged 25–35, recruited from the general population by announcements in local newspapers, and public meeting places. The study was conducted at the Department of Clinical Research at the University Hospital of Northern Norway (UNN), Tromsø, between 2000 and 2002 and has been described in detail elsewhere [14, 17, 38], we briefly summarize the methods. The participating women had to meet the following eligibility criteria: self-reported regular menstruation (cycle length: 22–38 days within the previous 3 months), no use of steroid contraceptives, pregnancy or lactation in the previous 6 months, no infertility, no history of gynecological disorders, and no chronic disorders (e.g. diabetes, hypo-/hyperthyroidism). At recruitment, subjects completed questionnaires and were interviewed by a trained nurse. Recall and memory-probing aids, including a lifetime calendar, were used to date specific life events [14, 17, 38]. These interviews including items on demographics, reproductive history, and lifestyle factors including: age at menarche, marital status, education, ethnicity, parity, physical activity, previous use of hormonal contraceptives, family history of cancer, smoking, and alcohol use [17, 38]. Birth weight obtained from the questionnaire and interview was also obtained by a linkage to the national Birth Registry. Two women were excluded from the current analyses due to missing mammographic data, resulting in 202 participants [38].

### Clinical parameters

All participants underwent clinical examinations within three specified intervals during a single menstrual cycle: (1) between days 1–5 after onset of bleeding; (2) days 7–12; and (3) days 21–25. Height was measured to the nearest 0.5 cm and weight to the nearest 0.1 kg on a regularly calibrated electronic scale. Body mass index (BMI) for our analysis was calculated as weight in kilograms per height in square meter (kg/m^2^) [38] using data from the first visit. Waist circumference (WC) was measured to the nearest 0.5 cm, 2.5 cm above the umbilicus. During the second visit a whole body scan was obtained for the estimation of the total percentage of fat tissue, using dual-energy X-ray absorptiometry (DEXA; DPLX-L 2288, Lunar Radiation Corporation, Madison, WI, USA). The percentage of fat tissue was estimated using Lunar software [14, 17, 37].

### Assessment of 17β-estradiol in serum and in saliva

At all three scheduled visits overnight fasting serum concentrations of 17β-estradiol were measured in fresh sera using a direct immunometric assay (Immuno-1; Bayer Diagnostics, Norway) at the Department of Clinical Chemistry, UNN [38]. The sensitivity for estradiol was 0.01 nmol/L, and the coefficient of variation (CV) was 3.9 %.

The participants self-collected daily morning saliva samples during the course of a whole menstrual cycle in order to assess the daily bioavailable fraction of 17β-estradiol. Sampling started on the first morning of menstrual bleeding and was conducted according to previously established and validated collection protocols [38, 43]. Samples were sent to the Reproductive Ecology Laboratory at Harvard University where they were stored at −70 °C until analysis. 17β-estradiol concentrations were measured in each saliva sample using 125I- labeled RIA kits (#39100, Diagnostic Systems Laboratories, Webster, TX, USA). All samples were run in duplicate, and samples from a single participant were run within the same assay batch. CVs were calculated based on the high and low value pools included in each assay [14, 17, 37].

Following estradiol assay, all cycles were aligned based on the identification of the mid-cycle drop in salivary 17 β-estradiol concentration (hereafter designated cycle day 0), which provides a reasonable estimate of the day of ovulation [17, 43, 44]. Two measures of 17β-estradiol were calculated for all participants: mean cycle-long concentration and mean mid-cycle (day −7 to +6) concentrations, as well as using daily levels of salivary 17β-estradiol. The mid-cycle 17β-estradiol drop could not be identified for 14 women, hence their cycles could not be aligned and they were omitted from the statistical analysis [17].

### Single-nucleotide polymorphism selection and genotyping

We analysed genetic polymorphisms in *ESR1*, 6q25, that encodes the estrogen receptor α. DNA was extracted from frozen whole blood using a MagAttract DNA Blood Mini M48 kit (QIAGEN, Valencia, CA, USA) by the Department of Medical Genetics, UNN. DNA was genotyped on the Golden Gate Platform (Illumina, San Diego, CA, USA) at the Fred Hutchinson Cancer Research Centre (Makar Lab), using the manufacturer’s protocol [17]. These methods have previously been described in detail [17]. In brief, 250 ng of genomic DNA was divided into aliquots in 96-well plates, processed accordingly and scanned on the Illumina iScan reader using BeadStudio software [17, 42].

We conducted a series of quality control procedures [45]. SNP call rates exceeded 99 % for this study, with 100 % concordance of blinded duplicates. The linkage disequilibrium select algorithm was employed to choose the tag SNPs via the Genome Variation Server [46, 47]. The SNPs were selected using an *r* ^2^ threshold of 0.8 and a minor allele frequency >5 %, representing variability in the white European population. Tag SNP coverage extended 2 kilobases (kb) upstream and 1 kb downstream of the gene, and 76 SNPs were covered. We further reduced the number of SNPs using power calculations and ended up with a final selection of 34 common SNPs with minor allele frequency >0.2. (Additional file 1: Table S1) None of the selected SNPs were monomorphic or significantly out of Hardy–Weinberg equilibrium [17].

### Mammograms and mammographic density

We obtained bilateral two-view mammograms from our subjects during the second scheduled visit (between cycle days 7 and 12) at the Centre for Breast Imaging, UNN, using a standard protocol [17, 38, 48]. The left craniocaudal mammograms were digitised and imported into a computerised mammographic density assessment programme (Madena) developed at the University of Southern California School of Medicine (Los Angeles, CA, USA) [38, 49, 50]. A single trained reader (G. Ursin) conducted all density measurements as follows: A region of interest (ROI) that included the entire breast was identified, (excluding the pectoralis muscle, prominent veins and fibrous strands). A tinting tool was used to highlight pixels representing dense areas of the mammograms within the ROI. The size of these dense areas (in square centimetres) was automatically calculated by the Madena software, giving a measure of absolute mammographic breast density [15, 38]. We then calculated percentage mammographic density as the ratio of absolute mammographic breast density to total breast area multiplied by 100 [37]. The mammograms were read in four batches, with an equal number of mammograms included in each batch. A duplicate reading of 26 randomly selected mammograms from two of the batches showed a Pearson’s correlation coefficient of 0.97. The reader was blinded to any characteristics of the study population [14, 17, 37, 38].

### Statistical methods

Descriptive characteristics were calculated by means (standard deviation) for continuous variables and percent for binary data. On the basis of the plausible biological mechanisms related to the estrogen metabolic pathway, we selected 34 SNPs in the *ESR1*, *6q25* gene for further analysis. These SNPs were coded as *AA* = 0 (major homozygous), *Aa* = 1 (heterozygous) and *aa* = 2 (minor homozygous) [17].

Mammographic density (absolute and percent) was considered continuously in our first set of models. We used multivariable linear regression models to assess the association between mammographic density phenotypes (absolute and percent mammographic density) as dependent variables and *ESR1* SNPs as ordinal independent variables.

Percent mammographic density and absolute mammographic density were used as both continuous and dichotomized variables, representing lower and higher density, using median values as cut-off points; Percent mammographic density (28.5 %), and absolute mammographic density (32.4 cm^2^) [16]. Previous studies of pre- and postmenopausal women have observed a 2–3 fold increase in breast cancer risk in women with percent mammographic density > 25 % [8, 51] and absolute mammographic density > 32 cm^2^. These observations along with our own observations related to estradiol levels [15, 37] support the comparison of women with above versus below median percent and absolute mammographic density [16]. Thus, mammographic density outcome variables were also used as dichotomized variables in logistic regression models where indicator variables of each SNP was included using aa as the reference level.

We considered several potential confounding factors known to be associated with mammographic density phenotypes, estrogen concentrations, and/or ESR1 variant. These included age (continuous), BMI (continuous), birth weight (continuous), age at menarche (continuous), parity (categorical), previous oral contraceptive use (categorical) and current smoking habits (categorical) [3]. Age, BMI, parity, current smoking habits, and previous oral contraceptive use were included as covariates in the final models. Salivary 17β-estradiol (continuous) and birth weight [13, 40], did not influence our estimates, and these were left out of our final model.

Based on plausible biological mechanisms and results from the first set of models, we selected eight SNPs (*rs3020364*, *rs2474148*, *rs12154178*, *rs2347867*, *rs6927072*, *rs2982712*, *rs3020407*, *rs9322335*) for further analyses in which we stratified the subjects based on median BMI (23.6 kg/m^2^). We again fitted multivariable linear regression models using the same set of covariates as previously specified, with the exception of BMI, to show how the relationship between percent mammographic density and the SNP genotypes might vary among different strata of women. More detailed stratification (i.e. to tertiles of body mass index) gave no additional information. Thus, BMI was used both as a continuous variable as well as a dichotomized one when we performed stratified analysis (comparing low BMI vs high BMI).

We used linear mixed models for repeated measures to study variations of daily salivary 17β-estradiol across the menstrual cycle, for subgroups of women with either major, minor homozygous or heterozygous genotypes for all eight SNPs, and adjusted for current smoking habits (yes/no), previous use of oral contraceptives (yes/no) as well as the same confounders as in the initial linear regression models, and stratified our data by median percent mammographic density (28.5 %). This revealed a suggested pattern for three of our eight SNPs (*rs3020364*, *rs2474148*, *rs2982712*).

All *P*-values were two-tailed and considered significant when the value was <0.05. The analyses were conducted with SPSS version 22.0 software (IBM, Armonk, NY, USA).

## Results

Table 1 provides selected general characteristics of the study participants. The participating women were on average 30.7 years old and had a mean BMI of 24.4 kg/m^2^. We found a mean salivary 17β-estradiol concentration of 17.9 pmol/l, mean percent mammographic density of 29.8 % (median 28.5 %), and mean absolute mammographic density of 34.7 cm^2^ (median 32.4 cm^2^). Common risk factors such as age, parity and body composition (BMI, WC, and total tissue fat) were inversely associated with mammographic density phenotypes. (Results not shown).

**Table 1:** Selected characteristics of the study population: The Norwegian EBBA-I study (*n* = 202)^a^

Characteristics	Total study population^b^
Age, years	30.7 (3.07)
Education, total years	16.1 (3.02)
Body composition^c^	
BMI, kg/m^2^	24.4 (3.77)
Waist, cm	79.5 (9.80)
Tissue fat, %^e^	34.2 (7.62)
Birth weight, g	3389 (561)
Reproductive factors	
Parity, No children	0.91 (1.13)
Time since last birth among parous, years	4.72 (3.07)
Age at menarche, years	13.1 (1.36)
Cycle length, days	28.2 (3.17)
Follicular phase length, days	14.9 (1.73)
Luteal phase length, days	13.45 (1.73)
Salivary hormones^d^	
Overall average 17β-estradiol, pmol/l	17.9 (8.79)
Overall average progesterone, pmol/l	130.2 (68.3)
Serum hormones^e^	
Estradiol, pmol/l	146.7 (61.6)
Progesterone, nmol/l	4.83 (6.29)
Lifestyle factors	
Previous use of oral contraceptives, %	82.7
Leisure time, MET h/week	57.6 (88.6)
Alcohol intake, units per week	2.89 (3.38)
Current smokers, %	22.1
Mammograms^f^	
Percent mammographic density, %	29.8 (19.0)
Absolute mammographic density, cm^2^	34.7 (23.4)

Abbreviations; *BMI* body mass index, *EBBA-1* The Norwegian Energy Balance and Breast cancer Aspects Study 1

^a^Numbers may vary due to missing information

^b^Values are mean (SD) or percent

^c^Measurements at day 1–5 after onset of menstrual cycle

^d^Daily saliva samples throughout an entire menstrual cycle

^e^Serum samples at day 7–12 (mid-cycle phase)

^f^Mammograms and total tissue fat (DEXA) were taken at day 7–12 (mid-cycle phase) after onset of the menstrual cycle

In our multiple linear regression models we found that having the minor homozygous allele was associated with decreased percent mammographic density for eight of our 34 SNPs: *rs3020364* (*p*-value 0.01), *rs2474148* (*p*-value 0.023), *rs12154178* (*p*-value 0.026), *rs2347867* (*p*-value 0.002), *rs6927072* (*p*-value 0.007), *rs2982712 p*-value 0.006), *rs3020407* (*p*-value 0.020), *rs9322335* (*p*-value 0.024) (Table 2). For all but two (*rs2347867*, *rs9322335*) of these eight SNPs this association was seen in lean, but not in heavier women when we dichotomized our data by median split of BMI (Lean: ≤23.6, Heavy > 23.6). With the exception of *rs9322335* (*p*-value 0.031), we did not observe similar associations in relation to absolute mammographic density (Additional file 2: Table S2).

**Table 2:** The linear association between the selected SNPs in the ESR1 region and percent mammographic density

SNPs	BMI variable	β-value	95 % CI^a^	*P*-value
*rs3020364*	Ungrouped^b^	−3.86	(−6.78, −0.95)	0.010
	BMI median split^c^			
	Low	−6.17	(−11.1, −1.29)	0.014
	High	−3.61	(−7.40, 0.18)	0.062
*rs2474148*	Ungrouped^b^	−3.37	(−6.26, −0.48)	0.023
	BMI median split^c^			
	Low	−4.10	(−9.04, 0.85)	0.103
	High	−4.60	(−8.32, −0.89)	0.016
*rs12154178*	Ungrouped^b^	−3.27	(−6.15, −0.39)	0.026
	BMI median split^c^			
	Low	−5.93	(−19.6, −1.29)	0.013
	High	−3.29	(−7.02, 0.45)	0.084
*rs2347867*	Ungrouped^b^	−4.24	(−6.97, −1.52)	0.002
	BMI median split^c^			
	Low	−5.94	(−10.2, −1.71)	0.006
	High	−4.34	(−8.09, −0.60)	0.024
*rs6927072*	Ungrouped^b^	−3.77	(−6.48, −1.06)	0.007
	BMI median split^c^			
	Low	−5.57	(−9.87, −1.24)	0.012
	High	−4.30	(−7.95, −0.65)	0.022
*rs2982712*	Ungrouped^b^	−3.89	(−6.67, −1.11)	0.006
	BMI median split^c^			
	Low	−6.18	(−10.7, −1.62)	0.008
	High	−3.02	(−6.69, 0.64)	0.104
*rs3020407*	Ungrouped^b^	−3.25	(−5.98, −0.51)	0.020
	BMI median split^c^			
	Low	−5.23	(−9.55, −0.91)	0.180
	High	−3.27	(−6.91, 0.37)	0.078
*rs9322335*	Ungrouped^b^	−3.46	(−6.46, −0.46)	0.24
	BMI median split^c^			
	Low	−5.61	(−10.5, −0.73)	0.025
	High	−2.71	(−6.70, 1.29)	0.182

^a^Confidence Interval

^b^Multivariable linear regression, adjusted by age, BMI, age at menarche and parity

^c^Multivariable linear regression, adjusted by age, age at menarche and parity. BMI median split at 23.64 kg/m^2^

^c^Only Single Nucleotide Polymorphisms (SNPs) with one or more two-tailed *p*-values < 0.05 represented (8/34 selected SNPs with Minor allele frequency (MAF) > 0.2)

The frequencies of genotypes of our selected SNPs in the study population were similar to those recorded in HapMap (Table 3). For seven out of eight SNPs, the odds of above-median percent mammographic density (>28.5 %) were 3–6 times higher among women with major homozygous genotypes compared to women with minor homozygous genotypes: *rs3020364*: OR 6.46 (*p*-value 0.009), *rs2474148*: OR 4.23 (*p*-value 0.028), *rs12154178*: OR 5.44 (*p*-value 0.014), *rs2347867*: OR 3.38 (*p*-value 0.030), *rs6927072*: OR 3.44 (*p*-value 0.028), *rs2982712*: OR 3.97 (*p*-value 0.013), *rs3020407*: OR 3.97 (*p*-value 0.024). When comparing the minor homozygous genotypes to the heterozygous, the odds of above median mammographic were higher for the heterozygous, but only significant for *rs2982712* (OR: 3.48, *p*-value 0.014) (Table 3).

**Table 3:** Selected SNP characteristics; Location, minor allele frequencies and adjusted Odds Ratio (OR) of above-median percent mammographic density (>28.5 %) by genotypes

SNP^a^	Location	Alleles	MAF^b^	Genotype	OR	95 % CI^c^	*P*-value
			EBBA-1 (HapMap)				
*rs302064*	Intron	A > G	0.368 (0.39)				
				aa	1.0	Ref	Ref
				Aa	3.74	0.95,14.6	0.059
				AA	6.46	1.61, 25.9	0.009
*rs2474148*	Intron	G > T	0.352 (0.35)				
				aa	1.0	Ref	Ref
				Aa	2.01	0.55, 7.39	0.29
				AA	4.23	1.17, 15.6	0.028
*rs12154178*	Intron	C < A	0.312 (0.26)				
				aa	1.0	Ref	Ref
				Aa	3.92	0.98, 7.39	0.054
				AA	5.44	1.42, 20.9	0.014
*rs2347867*	Intron	G > A	0.365 (0.30)				
				aa	1.0	Ref	Ref
				Aa	1.09	0.36, 3.29	0.879
				AA	3.38	1.23, 10.1	0.030
*rs6927072*	Intron	T > G	0.359 (0.28)				
				aa	1.0	Ref	Ref
				Aa	1.80	0.58, 5.56	0.309
				AA	3.44	1.15, 10.3	0.028
*rs2982712*	Intron	C < T	0.297 (−)				
				aa	1.0	Ref	Ref
				Aa	3.48	1.29, 9.36	0.014
				AA	3.97	1.34, 11.7	0.013
*rs3020407*	Intron	T < C	0.455 (0.47)				
				aa	1.0	Ref	Ref
				Aa	1.74	0.57, 10.3	0.331
				AA	3.47	1.17, 10.3	0.024
*rs9322335*	Intron	G > A	0.327 (0.27)				
				aa	1.0	Ref	Ref
				Aa	1.03	0.27, 3.88	0.969
				AA	1.77	0.49, 6.40	0.388

Odds ratio from logistic regression model adjusted for age, age at menarche, BMI and parity

^a^ *SNP* Single Nucleotide Polymorphisms

^b^ *MAF* Minor allele frequency

^c^ *CI* Confidence Interval

**Fig. 1:**
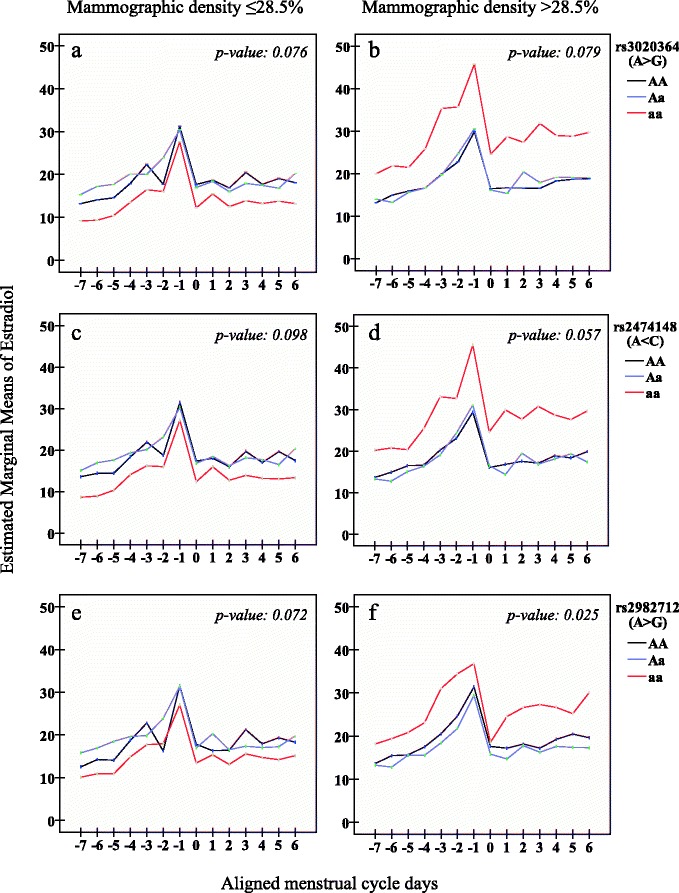
Salivary 17 β -estradiol levels across menstrual cycles for *rs3020364*, *rs2474148*, *rs2982712*. All analyses have used linear mixed models for repeated measures adjusted for age, age at menarche, parity, body mass index, current smoking and previous oral contraceptives. 95 % confidence intervals were removed for clarity. aa = minor homozygous genotype, Aa = heterozygous genotype, AA = major homozygous genotype. Mean 17β-estradiol levels by genotypes: **a** *rs3020364* and low percent mammographic density (<28.5 %): aa (*n* = 23);14.0 pmol/L, Aa(*n* = 44); 19.2 pmol/L, AA(*n* = 28); 18.5. **b** *rs3020364* and high percent mammographic density (≥28.5 %): aa (*n* = 21);29.0 pmol/L, Aa(*n* = 41); 18.7 pmol/L, AA(*n* = 45); 18.3. **c** *rs2474148* and low percent mammographic density (<28.5 %): aa (*n* = 21);14.0 pmol/L, Aa(*n* = 44); 19.1 pmol/L, AA(*n* = 30); 18.4. **d** *rs2474148* and high percent mammographic density (≥28.5 %): aa (*n* = 5);28.4 pmol/L, Aa(*n* = 35); 18.2 pmol/L, AA(*n* = 51); 18.5. **e** *rs2982712* and low percent mammographic density (<28.5 %): aa (*n* = 31);15.1 pmol/L, Aa(*n* = 42); 19.4pmol/L, AA(*n* = 22); 18.4. **f** *rs2982712* and high percent mammographic density (≥28.5 %): aa (*n* = 10);25.9 pmol/L, Aa(*n* = 50); 17.4 pmol/L, AA(*n* = 31); 19.2

When examining the 17β-estradiol concentrations throughout the menstrual cycle for women with high and low percent mammographic density separately (median split of percent mammographic density) using linear mixed models we found a striking, albeit only borderline significant pattern for 3 of our 8 SNPs; the level of 17β-estradiol for women with the minor aa genotype for *rs3020364*, *rs24744148* and *rs2982712* were lower throughout the cycle for women with low (<28.5 %) percent mammographic density and higher throughout the cycle for women with high (>28.5 %) percent mammographic density, when compared to women with the major AA genotype (Fig. 1) We observed a 24.3 % lower level of mean 17β- estradiol throughout a menstrual cycle in women with low mammographic density (<28.5 %) with minor genotype *aa* of *rs3020364* compared to those with major genotype *AA* (Fig. 1a, *p*-value 0.076) and in women with high mammographic density and minor genotype aa of the same SNP we observed a 58 % higher level of mean 17β-estradiol throughout the cycle compared with women with major genotype AA. (Fig. 1b: *p*-value 0.079). Fig. 1c-f show a similar pattern for *rs2474148* and *rs2982714*, results in Fig. 1f are significant (*p*-value 0.025).

## Discussion

We have assessed the association between 34 polymorphisms in the ESR1 gene with mammographic density phenotypes and daily cycling estradiol in healthy, premenopausal, Norwegian women. Our results support an association between 8 (*rs3020364*, *rs2474148*, *rs12154178*, *rs2347867*, *rs6927072*, *rs2982712*, *rs3020407*, *rs9322335*) studied SNPs and percent mammographic density, and one SNP (rs9322335) also associated with absolute mammographic density. For all but two of these SNPs (rs2347867, rs9322335) this inverse linear association was maintained in lean (BMI ≤23.6 kg/m^2^), but not in heavier women (BMI >23.6 kg/m^2^). We also suggest, based on results of borderline significance that the level of 17β-estradiol for women with the minor aa genotype for *rs3020364*, *rs24744148* and *rs2982712* were lower throughout the cycle for women with low (<28.5 %) percent mammographic density and higher throughout the cycle for women with high (>28.5 %) percent mammographic density, when compared to women with the major AA genotype.

All 8 SNPs associated with mammographic density in the present study are intronic. It is not yet clear why and how noncoding SNPs influence the gene activity [17], but one suggested mechanism is that intronic SNPs may regulate gene expression through endogenous transacting factors, epigenetics and chromosome conformation [52]. Previous GWAS studies have shown intronic SNPs to be important breast cancer risk loci [53]. This does not imply causality, but may aid in the identification of novel susceptibility loci [17]. The ERα-receptor mediates estrogen action by regulating gene transcription, triggering the mRNA-synthesis and consequently the production of proteins that produce the physiological hormone effects [54]. The expression of ERα seems to be linked to breast cell proliferation as well as to be increased during tumorigenesis [55].

Since one of the mechanisms by which estrogen promotes the proliferation of both normal and neoplastic breast epithelial cells is through its binding to ERα, and that the content of ERα in breast tissue is associated with increased breast cell proliferation [56, 57], a modification of the expression of this gene could influence mammographic density or breast cancer risk. Indeed, a study among postmenopausal women found a statistically significant increased protein expression of ERα in normal epithelium of women with high mammographic density compared to those with low density [58]. To our knowledge, there are no studies related to *ESR1* SNPs, daily levels of estrogens throughout an entire menstrual cycle and mammographic density phenotypes in premenopausal women, and published results on the specific SNPs analysed in our current study do not exist.

The possible associations between SNPs in the ER genes and mammographic density are still under debate [25, 26, 29–31]. Some in vitro studies have shown that the expression of ERα in breast cancer cells may be down regulated by estradiol [59]. Recently published results from a study of 3,872 common genetic variants across three ESR1 loci with subjects from three international consortia found evidence for at least five independent causal variants associated with phenotype sets, mammographic density being one. We found no evidence of correspondence between our SNPs and the causal variants identified, but this may be explained by the fact that due to our small sample size we identified 34 SNPs with a sufficient MAF >0.2, while the newly published article could include the genotypes of MAF > 0.02 [60].

Thus far, research has mostly focused on SNPs *rs2234693* (*Pvu*II) or *rs9340799* (*Xba*I). The impact of *Pvu*II and *Xba*I variant alleles on *ESR1* transcription is not well established. A recent meta-analysis including 11 studies identified reduced breast cancer risk for those carrying homozygous the minor allele of SNP *rs2234693*, while no association was observed for SNP *rs9340799* [61]. Others have suggested that these variants are associated with reduced breast cancer risk among women with lower levels of estrogen [62, 63], and these findings complement those emerging from some of the studies on mammographic density: Among a population of pre- and postmenopausal women, the minor allele of the *rs9340799* SNP was associated with decreased mammographic density, while no association was found for ERα *rs2234693* [29]. By contrast, others have found that premenopausal women with the homozygous minor allele of SNP *rs2234693* (ERα) have higher mammographic density. Similar associations have been suggested for *rs9340799* [25]. It is, therefore, plausible that estrogen concentrations may influence the relationship between susceptibility genes and mammographic density. This suggests a biological rationale for our findings of different estrogen levels throughout the menstrual cycle of genotype aa compared to AA when our population was split by percent mammographic density (Fig. 1). Our results suggest that having the minor homozygous allele is associated with decreased percent mammographic density, and the associations are stronger in lean women. However, no association has been observed within strata of estrogen-related factors (parity, hormonal derivatives used, age at menarche, BMI) among premenopausal women [26], nor in populations of premenopausal and postmenopausal women ([26, 30, 31]. Other investigated SNPs (*rs2228480*, *rs728524*, *rs3798577* and *rs2077647*) have shown no association with mammographic density in premenopausal and/or postmenopausal populations [25, 26].

Several of our findings are novel. To our knowledge, no other studies have focused on the associations between mammographic density phenotypes and these 8 SNPs in the *ESR1* gene. In a large case–control study, 41 independent breast cancer susceptibility variants were discovered using a custom genotyping array designed, in part, by the Breast Cancer Association Consortium [64]. Of these variants, a recent report found novel associations between breast cancer SNPs in *6q25* including *rs9383938* in the *ESR1* region with a volumetric measure of mammographic density in 5000 Swedish women [65]. The marker of density consortium (MODE) recently identified nine loci associated with an area-based mammographic density one of which is *ESR1* [66, 67].

Specific SNPs located in genes of this pathway could directly or indirectly lead to variations that may have effects on breast cancer risk. Also, estrogens are known to regulate the activity of several enzymes and receptors in the estrogen pathway [59, 68–70] Therefore, the relation between SNPs of such genes and breast cancer risk could vary among strata of women based on their estradiol levels [71]. However, no overall linear association was found between our SNPs and estradiol.

The strengths of our study include daily measurements of salivary concentrations of unbound, bioactive 17β-estradiol collected across an entire menstrual cycle [72], as well as following strict procedures [38], and validated methods [44]. Among ovulating premenopausal women, the estrogen levels vary considerably throughout the menstrual cycle [14]. Using daily salivary samples, we were able to measure the free biologically active form of estrogen, which is considered to be the ideal among ovulating premenopausal women [14, 44, 73]. Previous research has indicated that multiple measurements of unbound bioavailable levels probably give us a picture of the real and cumulative estrogen exposure over time [7, 74]. Thus, we were able to capture the continuous estrogen exposure of the included women. We also included standardized repeated serum hormone levels. We have previously observed, based on the same study population, that daily levels of salivary estradiol may be associated with known breast cancer risk factors including mammographic density phenotypes [15, 37]. Moreover, mammographic density measures were obtained within a narrow time frame during the late follicular phase (days 7–12), thereby avoiding the bias of variation in mammographic density throughout the menstrual cycle [75]. The validated computer-assisted method used to quantify mammographic density has been shown to give a superior prediction of breast cancer risk compared to qualitative methods [49]. A single experienced blinded reader read all mammograms, and the assessed mammographic density was negatively associated with age, BMI, and parity [76].

The relatively small sample size of the current study must be considered as one of the limitations warranting attention, and underlines the need for further and larger studies. The study design was cross-sectional, and population stratification can be of concern in this type of study [77], but our study population consisted of Norwegian/Caucasian women with only a ten-year age span.

Based on the biological hypothesis that polymorphisms in the ESR1 gene may influence 17β-estradiol levels and mammographic phenotypes, we examined a limited number of SNPs [17]. Even though there is a risk of false-positive results/type I errors in multiple testing, our analysis are based on carefully considered plausible biological mechanisms, and an a priori hypothesis of difference rather than a universal null-hypothesis. Hence, we considered the increased likelihood of type II errors and the risk of deeming truly interesting difference non-significant when introducing Bonferroni-adjustments just as important.

In recent studies, the immunoassay methods used in the present study is most often replaced by Liquid Chromatography (LC) - Mass Spectrometry (MS/MS), which is considered a more efficient method of analysing salivary hormones with higher specificity and sensitivity. However, studies on estradiol measurements have demonstrated a high correlation between LC/MS-MS and immunoassays of 0.969 [15, 17, 78].

## Conclusions

The *6q25* region is important in the etiology of breast cancer among women with dense breasts [79], but its putative functions are still undefined. Identifying genetic variants that are associated with both breast cancer risk and mammographic density measures that predict breast cancer has the potential to reveal underlying biological pathways that explain the associations between those mammographic measures and cancer. Our data provide additional evidence for the relationship between genetic variants, mammographic density phenotypes and estradiol. We hypothesize that *ESR1* may play a role in the relationship between dense breast tissue and estradiol levels, and therefore, the risk of breast cancer.

## Additional files

Additional file 1: Table S1. Location, switch and population genotype frequencies of selected SNPs in the ESR1 gene. (DOCX 25 kb)
Additional file 2: Table S2. The linear association between the selected SNPs in the ESR1 region and absolute mammographic density. (DOC 47 kb)

## Abbreviations

BMI: Body mass index
CI: Confidence interval
CV: Coefficient of variance
EBBA-1: Norwegian Energy Balance and Breast cancer Aspects
ESR1: Estrogen receptor alpha gene
GWAS: Genome wide association study
MAF: Minor allele frequency
OR: Odds ratio
ROI: Region of interest
SNP: Single nucleotide polymorphism
UNN: University hospital of North Norway

## References

[1] Ursin G, Lillie EO, Lee E, Cockburn M, Schork NJ, Cozen W, Parisky YR, Hamilton AS, Astrahan MA, Mack T (2009). The relative importance of genetics and environment on mammographic density. Cancer Epidemiol Biomarkers Prev.

[2] Boyd NF, Guo H, Martin LJ, Sun L, Stone J, Fishell E, Jong RA, Hislop G, Chiarelli A, Minkin S (2007). Mammographic density and the risk and detection of breast cancer. N Engl J Med.

[3] Vachon CM, Kuni CC, Anderson K, Anderson VE, Sellers TA (2000). Association of mammographically defined percent breast density with epidemiologic risk factors for breast cancer (United States). Cancer Causes Control.

[4] Russo J, Russo IH (2004). Genotoxicity of steroidal estrogens. Trends Endocrinol Metab.

[5] Folkerd E, Dowsett M (2013). Sex hormones and breast cancer risk and prognosis. Breast.

[6] Key T, Appleby P, Barnes I, Reeves G, Endogenous H, Breast Cancer Collaborative G (2002). Endogenous sex hormones and breast cancer in postmenopausal women: reanalysis of nine prospective studies. J Natl Cancer Inst.

[7] Key TJ, Appleby PN, Reeves GK, Travis RC, Alberg AJ, Barricarte A, Berrino F, Krogh V, Endogenous H, Breast Cancer Collaborative G (2013). Sex hormones and risk of breast cancer in premenopausal women: a collaborative reanalysis of individual participant data from seven prospective studies. Lancet Oncol.

[8] Yaghjyan L, Colditz GA, Rosner B, Tamimi RM (2013). Mammographic breast density and subsequent risk of breast cancer in postmenopausal women according to the time since the mammogram. Cancer Epidemiol Biomarkers Prev.

[9] Boyd NF, Jensen HM, Cooke G, Han HL, Lockwood GA, Miller AB (2000). Mammographic densities and the prevalence and incidence of histological types of benign breast disease. Reference Pathologists of the Canadian National Breast Screening Study. Eur J Cancer Prev.

[10] McCormack VA, dos Santos SI (2006). Breast density and parenchymal patterns as markers of breast cancer risk: a meta-analysis. Cancer Epidemiol Biomarkers Prev.

[11] Pettersson A, Graff RE, Ursin G, Santos Silva ID, McCormack V, Baglietto L, Vachon C, Bakker MF, Giles GG, Chia KS et al. Mammographic density phenotypes and risk of breast cancer: a meta-analysis. J Natl Cancer Inst. 2014;106(5). doi:10.93/jnci/dju078.Review. 10.1093/jnci/dju078 PMC4568991 24816206

[12] Greendale GA, Reboussin BA, Slone S, Wasilauskas C, Pike MC, Ursin G (2003). Postmenopausal hormone therapy and change in mammographic density. J Natl Cancer Inst.

[13] McTiernan A, Martin CF, Peck JD, Aragaki AK, Chlebowski RT, Pisano ED, Wang CY, Brunner RL, Johnson KC, Manson JE (2005). Estrogen-plus-progestin use and mammographic density in postmenopausal women: Women’s Health Initiative randomized trial. J Natl Cancer Inst.

[14] Frydenberg H, Flote VG, Iversen A, Finstad SE, Furberg AS, Torjesen PA, Wilsgaard T, Schlichting E, Ellison PT, Ursin G (2014). Insulin-like growth factor-1, growth hormone, and daily cycling estrogen are associated with mammographic density in premenopausal women. Cancer Causes Control.

[15] Frydenberg H, Flote VG, Larsson IM, Barrett ES, Furberg AS, Ursin G, Wilsgaard T, Ellison PT, McTiernan A, Hjartaker A (2015). Alcohol consumption, endogenous estrogen and mammographic density among premenopausal women. Breast Cancer Res.

[16] Flote VG, Frydenberg H, Ursin G, Iversen A, Fagerland MW, Ellison PT, Wist EA, Egeland T, Wilsgaard T, McTiernan A (2015). High-density lipoprotein-cholesterol, daily estradiol and progesterone, and mammographic density phenotypes in premenopausal women. Cancer Prev Res (Phila).

[17] Flote VG, Furberg A, McTiernan A, Frydenberg H, Ursin G, Iversen A, Lofteroed T, Ellison PT, Wist EA, Egeland T (2014). Gene variations in oestrogen pathways, CYP19A1, daily 17ss-estradiol and mammographic density phenotypes in premenopausal women. Breast Cancer Res.

[18] Ursin G, Hovanessian-Larsen L, Parisky YR, Pike MC, Wu AH (2005). Greatly increased occurrence of breast cancers in areas of mammographically dense tissue. Breast Cancer Res.

[19] Gill JK, Maskarinec G, Pagano I, Kolonel LN (2006). The association of mammographic density with ductal carcinoma in situ of the breast: the Multiethnic Cohort. Breast Cancer Res.

[20] Rossouw JE, Anderson GL, Prentice RL, LaCroix AZ, Kooperberg C, Stefanick ML, Jackson RD, Beresford SA, Howard BV, Johnson KC (2002). Risks and benefits of estrogen plus progestin in healthy postmenopausal women: principal results From the Women’s Health Initiative randomized controlled trial. JAMA.

[21] Boyd NF, Melnichouk O, Martin LJ, Hislop G, Chiarelli AM, Yaffe MJ, Minkin S (2011). Mammographic density, response to hormones, and breast cancer risk. J Clin Oncol.

[22] Ahmed S, Thomas G, Ghoussaini M, Healey CS, Humphreys MK, Platte R, Morrison J, Maranian M, Pooley KA, Luben R (2009). Newly discovered breast cancer susceptibility loci on 3p24 and 17q23.2. Nat Genet.

[23] Fletcher O, Johnson N, Orr N, Hosking FJ, Gibson LJ, Walker K, Zelenika D, Gut I, Heath S, Palles C (2011). Novel breast cancer susceptibility locus at 9q31.2: results of a genome-wide association study. J Natl Cancer Inst.

[24] Yamamoto-Ibusuki M, Yamamoto Y, Fujiwara S, Sueta A, Yamamoto S, Hayashi M, Tomiguchi M, Takeshita T, Iwase H (2014). C6ORF97-ESR1 breast cancer susceptibility locus: influence on progression and survival in breast cancer patients. Eur J Hum Genet.

[25] Crandall CJ, Sehl ME, Crawford SL, Gold EB, Habel LA, Butler LM, Sowers MR, Greendale GA, Sinsheimer JS (2009). Sex steroid metabolism polymorphisms and mammographic density in pre- and early perimenopausal women. Breast Cancer Res.

[26] Dumas I, Diorio C (2010). Polymorphisms in genes involved in the estrogen pathway and mammographic density. BMC Cancer.

[27] Haiman CA, Hankinson SE, De Vivo I, Guillemette C, Ishibe N, Hunter DJ, Byrne C (2003). Polymorphisms in steroid hormone pathway genes and mammographic density. Breast Cancer Res Treat.

[28] Maskarinec G, Lurie G, Williams AE, Le Marchand L (2004). An investigation of mammographic density and gene variants in healthy women. Int J Cancer.

[29] van Duijnhoven FJ, Bezemer ID, Peeters PH, Roest M, Uitterlinden AG, Grobbee DE, van Gils CH (2005). Polymorphisms in the estrogen receptor alpha gene and mammographic density. Cancer Epidemiol Biomarkers Prev.

[30] van Duijnhoven FJ, Peeters PH, Warren RM, Bingham SA, Uitterlinden AG, van Noord PA, Monninkhof EM, Grobbee DE, van Gils CH (2006). Influence of estrogen receptor alpha and progesterone receptor polymorphisms on the effects of hormone therapy on mammographic density. Cancer Epidemiol Biomarkers Prev.

[31] Warren R, Skinner J, Sala E, Denton E, Dowsett M, Folkerd E, Healey CS, Dunning A, Doody D, Ponder B (2006). Associations among mammographic density, circulating sex hormones, and polymorphisms in sex hormone metabolism genes in postmenopausal women. Cancer Epidemiol Biomarkers Prev.

[32] Barzan D, Veldwijk MR, Herskind C, Li Y, Zhang B, Sperk E, Du WD, Zhang XJ, Wenz F (2013). Comparison of genetic variation of breast cancer susceptibility genes in Chinese and German populations. Eur J Hum Genet.

[33] Haiman CA, Bernstein L, Berg D, Ingles SA, Salane M, Ursin G (2002). Genetic determinants of mammographic density. Breast Cancer Res.

[34] Li J, Eriksson L, Humphreys K, Czene K, Liu J, Tamimi RM, Lindstrom S, Hunter DJ, Vachon CM, Couch FJ (2010). Genetic variation in the estrogen metabolic pathway and mammographic density as an intermediate phenotype of breast cancer. Breast Cancer Res.

[35] Stone J, Gurrin LC, Byrnes GB, Schroen CJ, Treloar SA, Padilla EJ, Dite GS, Southey MC, Hayes VM, Hopper JL (2007). Mammographic density and candidate gene variants: a twins and sisters study. Cancer Epidemiol Biomarkers Prev.

[36] Takata Y, Maskarinec G, Le Marchand L (2007). Breast density and polymorphisms in genes coding for CYP1A2 and COMT: the Multiethnic Cohort. BMC Cancer.

[37] Iversen A, Frydenberg H, Furberg AS, Flote VG, Finstad SE, McTiernan A, Ursin G, Wilsgaard T, Ellison PT, Jasienska G (2015). Cyclic endogenous estrogen and progesterone vary by mammographic density phenotypes in premenopausal women. Eur J Cancer Prev.

[38] Furberg AS, Jasienska G, Bjurstam N, Torjesen PA, Emaus A, Lipson SF, Ellison PT, Thune I (2005). Metabolic and hormonal profiles: HDL cholesterol as a plausible biomarker of breast cancer risk. The Norwegian EBBA Study. Cancer Epidemiol Biomarkers Prev.

[39] Iversen A, Thune I, McTiernan A, Emaus A, Finstad SE, Flote V, Wilsgaard T, Lipson SF, Ellison PT, Jasienska G (2011). Ovarian hormones and reproductive risk factors for breast cancer in premenopausal women: the Norwegian EBBA-I study. Hum Reprod.

[40] Emaus A, Veierod MB, Furberg AS, Espetvedt S, Friedenreich C, Ellison PT, Jasienska G, Andersen LB, Thune I (2008). Physical activity, heart rate, metabolic profile, and estradiol in premenopausal women. Med Sci Sports Exerc.

[41] Finstad SE, Emaus A, Tretli S, Jasienska G, Ellison PT, Furberg AS, Wist EA, Thune I (2009). Adult height, insulin, and 17beta-estradiol in young women. Cancer Epidemiol Biomarkers Prev.

[42] Iversen A, Thune I, McTiernan A, Makar KW, Wilsgaard T, Ellison PT, Jasienska G, Flote V, Poole EM, Furberg AS (2012). Genetic polymorphism CYP17 rs2486758 and metabolic risk factors predict daily salivary 17beta-estradiol concentration in healthy premenopausal Norwegian women. The EBBA-I study. J Clin Endocrinol Metab.

[43] Lipson SF, Ellison PT (1996). Comparison of salivary steroid profiles in naturally occurring conception and non-conception cycles. Hum Reprod.

[44] Ellison PT, Lipson SF (1999). Salivary estradiol--a viable alternative?. Fertil Steril.

[45] Passarelli MN, Phipps AI, Potter JD, Makar KW, Coghill AE, Wernli KJ, White E, Chan AT, Hutter CM, Peters U (2013). Common single-nucleotide polymorphisms in the estrogen receptor beta promoter are associated with colorectal cancer survival in postmenopausal women. Cancer Res.

[46] Carlson CS, Eberle MA, Rieder MJ, Yi Q, Kruglyak L, Nickerson DA (2004). Selecting a maximally informative set of single-nucleotide polymorphisms for association analyses using linkage disequilibrium. Am J Hum Genet.

[47] Thorisson GA, Smith AV, Krishnan L, Stein LD (2005). The International HapMap Project Web site. Genome Res.

[48] Bjurstam N, Bjorneld L, Warwick J, Sala E, Duffy SW, Nystrom L, Walker N, Cahlin E, Eriksson O, Hafstrom LO (2003). The Gothenburg Breast Screening Trial. Cancer.

[49] Ursin G, Astrahan MA, Salane M, Parisky YR, Pearce JG, Daniels JR, Pike MC, Spicer DV (1998). The detection of changes in mammographic densities. Cancer Epidemiol Biomarkers Prev.

[50] Ursin G, Ma H, Wu AH, Bernstein L, Salane M, Parisky YR, Astrahan M, Siozon CC, Pike MC (2003). Mammographic density and breast cancer in three ethnic groups. Cancer Epidemiol Biomarkers Prev.

[51] van Gils CH, Hendriks JH, Otten JD, Holland R, Verbeek AL (2000). Parity and mammographic breast density in relation to breast cancer risk: indication of interaction. Eur J Cancer Prev.

[52] Robbez-Masson LJ, Bodor C, Jones JL, Hurst HC, Fitzgibbon J, Hart IR, Grose RP (2013). Functional analysis of a breast cancer-associated FGFR2 single nucleotide polymorphism using zinc finger mediated genome editing. PLoS One.

[53] Milne RL, Burwinkel B, Michailidou K, Arias-Perez JI, Zamora MP, Menendez-Rodriguez P, Hardisson D, Mendiola M, Gonzalez-Neira A, Pita G (2014). Common non-synonymous SNPs associated with breast cancer susceptibility: findings from the Breast Cancer Association Consortium. Hum Mol Genet.

[54] Bai Z, Gust R (2009). Breast cancer, estrogen receptor and ligands. Arch Pharm (Weinheim).

[55] Brandenberger AW, Tee MK, Jaffe RB (1998). Estrogen receptor alpha (ER-alpha) and beta (ER-beta) mRNAs in normal ovary, ovarian serous cystadenocarcinoma and ovarian cancer cell lines: down-regulation of ER-beta in neoplastic tissues. J Clin Endocrinol Metab.

[56] Russo J, Ao X, Grill C, Russo IH (1999). Pattern of distribution of cells positive for estrogen receptor alpha and progesterone receptor in relation to proliferating cells in the mammary gland. Breast Cancer Res Treat.

[57] Russo J, Russo IH (2006). The role of estrogen in the initiation of breast cancer. J Steroid Biochem Mol Biol.

[58] Lundstrom E, Sahlin L, Skoog L, Hagerstrom T, Svane G, Azavedo E, Sandelin K, von Schoultz B (2006). Expression of Syndecan-1 in histologically normal breast tissue from postmenopausal women with breast cancer according to mammographic density. Climacteric.

[59] Borras M, Hardy L, Lempereur F, el Khissiin AH, Legros N, Gol-Winkler R, Leclercq G (1994). Estradiol-induced down-regulation of estrogen receptor. Effect of various modulators of protein synthesis and expression. J Steroid Biochem Mol Biol.

[60] Dunning AM, Michailidou K, Kuchenbaecker KB, Thompson D, French JD, Beesley J, Healey CS, Kar S, Pooley KA, Lopez-Knowles E (2016). Breast cancer risk variants at 6q25 display different phenotype associations and regulate ESR1, RMND1 and CCDC170. Nat Genet.

[61] Li N, Dong J, Hu Z, Shen H, Dai M (2010). Potentially functional polymorphisms in ESR1 and breast cancer risk: a meta-analysis. Breast Cancer Res Treat.

[62] Shin A, Kang D, Nishio H, Lee MJ, Park SK, Kim SU, Noh DY, Choe KJ, Ahn SH, Hirvonen A (2003). Estrogen receptor alpha gene polymorphisms and breast cancer risk. Breast Cancer Res Treat.

[63] Onland-Moret NC, van Gils CH, Roest M, Grobbee DE, Peeters PH (2005). The estrogen receptor alpha gene and breast cancer risk (The Netherlands). Cancer Causes Control.

[64] Michailidou K, Hall P, Gonzalez-Neira A, Ghoussaini M, Dennis J, Milne RL, Schmidt MK, Chang-Claude J, Bojesen SE, Bolla MK (2013). Large-scale genotyping identifies 41 new loci associated with breast cancer risk. Nat Genet.

[65] Brand JS, Humphreys K, Thompson DJ, Li J, Eriksson M, Hall P, Czene K. Volumetric mammographic density: heritability and association with breast cancer susceptibility loci. J Natl Cancer Inst. 2014;106(12). doi:10.093/jnci/dju334.Print2014dec. 10.1093/jnci/dju334 25376863

[66] Lindstrom S, Thompson DJ, Paterson AD, Li J, Gierach GL, Scott C, Stone J, Douglas JA, Dos-Santos-Silva I, Fernandez-Navarro P (2014). Genome-wide association study identifies multiple loci associated with both mammographic density and breast cancer risk. Nat Commun.

[67] Lindstrom S, Vachon CM, Li J, Varghese J, Thompson D, Warren R, Brown J, Leyland J, Audley T, Wareham NJ (2011). Common variants in ZNF365 are associated with both mammographic density and breast cancer risk. Nat Genet.

[68] Tsuchiya Y, Nakajima M, Kyo S, Kanaya T, Inoue M, Yokoi T (2004). Human CYP1B1 is regulated by estradiol via estrogen receptor. Cancer Res.

[69] Tsuchiya Y, Nakajima M, Yokoi T (2005). Cytochrome P450-mediated metabolism of estrogens and its regulation in human. Cancer Lett.

[70] Jiang H, Xie T, Ramsden DB, Ho SL (2003). Human catechol-O-methyltransferase down-regulation by estradiol. Neuropharmacology.

[71] Dumas I, Diorio C (2011). Estrogen pathway polymorphisms and mammographic density. Anticancer Res.

[72] Gann PH, Giovanazzi S, Van Horn L, Branning A, Chatterton RT (2001). Saliva as a medium for investigating intra- and interindividual differences in sex hormone levels in premenopausal women. Cancer Epidemiol Biomarkers Prev.

[73] Bellem A, Meiyappan S, Romans S, Einstein G (2011). Measuring estrogens and progestagens in humans: an overview of methods. Gend Med.

[74] Schoemaker MJ, Folkerd EJ, Jones ME, Rae M, Allen S, Ashworth A, Dowsett M, Swerdlow AJ (2014). Combined effects of endogenous sex hormone levels and mammographic density on postmenopausal breast cancer risk: results from the Breakthrough Generations Study. Br J Cancer.

[75] Morrow M, Chatterton RT, Rademaker AW, Hou N, Jordan VC, Hendrick RE, Khan SA (2010). A prospective study of variability in mammographic density during the menstrual cycle. Breast Cancer Res Treat.

[76] Byrne C, Schairer C, Wolfe J, Parekh N, Salane M, Brinton LA, Hoover R, Haile R (1995). Mammographic features and breast cancer risk: effects with time, age, and menopause status. J Natl Cancer Inst.

[77] Rothman KJ, Greenland S (1998). Modern epidemiology.

[78] Holst JP, Soldin OP, Guo T, Soldin SJ (2004). Steroid hormones: relevance and measurement in the clinical laboratory. Clin Lab Med.

[79] Holst F, Stahl PR, Ruiz C, Hellwinkel O, Jehan Z, Wendland M, Lebeau A, Terracciano L, Al-Kuraya K, Janicke F (2007). Estrogen receptor alpha (ESR1) gene amplification is frequent in breast cancer. Nat Genet.

