# Supplementary material for: Polymorphisms in the estrogen receptor alpha gene (ESR1), daily cycling estrogen and mammographic density phenotypes
Source: BMC Cancer. 2016 Oct 7;16:776. doi: 10.1186/s12885-016-2804-1 (PMC5055696; doi:10.1186/s12885-016-2804-1)
Supplement: Additional file 1: Table S1. — Location, switch and population genotype frequencies of selected SNPs in the ESR1 gene. (DOCX 25 kb) [file 12885_2016_2804_MOESM1_ESM.docx]

**Additional file 1: Table S1. Location, switch and population genotype frequencies of selected SNPs in the *ESR1* gene.**

| **SNP** | **Location** | **Switch** | **Genotype** | **EBBA-1** | **Caucation** | **Chinese** | **African** |
| --- | --- | --- | --- | --- | --- | --- | --- |
| ***rs3020364*** | Intron | A>G | aa | 0.109 |  |  |  |
|  |  |  | Aa | 0.521 |  |  |  |
|  |  |  | AA | 0.37 |  |  |  |
|  |  |  | MAF | 0.368 | 0.39 | 0.46 | 0.29 |
| ***rs2474148*** | Intron | G>T | aa | 0.402 |  |  |  |
|  |  |  | Aa | 0.492 |  |  |  |
|  |  |  | AA | 0.105 |  |  |  |
|  |  |  | MAF | 0.352 | 0.35 | 0.47 | 0.34 |
| ***rs1514348*** | Intron | T>G | aa | 0.113 |  |  |  |
|  |  |  | Aa | 0.451 |  |  |  |
|  |  |  | AA | 0.436 |  |  |  |
|  |  |  | MAF | 0.338 | 0.40 | 0.46 | 0.35 |
| ***rs827423*** | Intron | G<A | aa | 0.188 |  |  |  |
|  |  |  | Aa | 0.425 |  |  |  |
|  |  |  | AA | 0.387 |  |  |  |
|  |  |  | MAF | 0.4 | 0.44 | 0.36 | 0.39 |
| ***rs3020318*** | Intron | T>C | aa | 0.513 |  |  |  |
|  |  |  | Aa | 0.37 |  |  |  |
|  |  |  | AA | 0.111 |  |  |  |
|  |  |  | MAF | 0.301 | 0.28 | 0.33 | 0.10 |
| ***rs1884054*** | Intron | C>A | aa | 0.117 |  |  |  |
|  |  |  | Aa | 0.38 |  |  |  |
|  |  |  | AA | 0.504 |  |  |  |
|  |  |  | MAF | 0.306 | 0.29 | 0.32 | 0.08 |
| ***rs1801132*** | Coding-synonymous | G<C | aa | 0.609 |  |  |  |
|  |  |  | Aa | 0.338 |  |  |  |
|  |  |  | AA | 0.053 |  |  |  |
|  |  |  | MAF | 0.222 | 0.18 | 0.48 | 0.06 |
| ***rs6912184*** | Intron | G<A | aa | 0.053 |  |  |  |
|  |  |  | Aa | 0.336 |  |  |  |
|  |  |  | AA | 0.611 |  |  |  |
|  |  |  | MAF | 0.22 | 0.22 | 0.45 | 0.20 |
| ***rs12154178*** | Intron | C<A | aa | 0.109 |  |  |  |
|  |  |  | Aa | 0.408 |  |  |  |
|  |  |  | AA | 0.483 |  |  |  |
|  |  |  | MAF | 0.312 | 0.26 | 0.37 | 0.32 |
| ***rs2347867*** | Intron | G>A | aa | 0.135 |  |  |  |
|  |  |  | Aa | 0.459 |  |  |  |
|  |  |  | AA | 0.406 |  |  |  |
|  |  |  | MAF | 0.365 | 0.30 | 0.21 | 0.23 |
| ***rs1709182*** | Intron | T>C | aa | 0.079 |  |  |  |
|  |  |  | Aa | 0.429 |  |  |  |
|  |  |  | AA | 0.492 |  |  |  |
|  |  |  | MAF | 0.293 | 0.36 | 0.02 | 0.06 |
| ***rs9340799*** | Intron | A>G | aa | 0.068 |  |  |  |
|  |  |  | Aa | 0.395 |  |  |  |
|  |  |  | AA | 0.538 |  |  |  |
|  |  |  | MAF | 0.265 | 0.30 | 0.22 | 0.18 |
| ***rs532010*** | Intron | A>G | aa | 0.087 |  |  |  |
|  |  |  | Aa | 0.457 |  |  |  |
|  |  |  | AA | 0.457 |  |  |  |
|  |  |  | MAF | 0.314 | 0.31 | 0.36 | 0.45 |
| ***rs722208*** | Intron | A>G | aa | 0.079 |  |  |  |
|  |  |  | Aa | 0.498 |  |  |  |
|  |  |  | AA | 0.423 |  |  |  |
|  |  |  | MAF | 0.327 | 0.27 | 0.46 | 0.39 |
| ***rs3020314*** | Intron | C>T | aa | 0.132 |  |  |  |
|  |  |  | Aa | 0.362 |  |  |  |
|  |  |  | AA | 0.506 |  |  |  |
|  |  |  | MAF | 0.312 | 0.29 | 0.18 | 0.24 |
| ***rs3020403*** | Intron | G>C | aa | 0.121 |  |  |  |
|  |  |  | Aa | 0.34 |  |  |  |
|  |  |  | AA | 0.54 |  |  |  |
|  |  |  | MAF | 0.289 | 0.23 | 0.33 | 0.09 |
| ***rs2982896*** | Intron | C>T | aa | 0.53 |  |  |  |
|  |  |  | Aa | 0.414 |  |  |  |
|  |  |  | AA | 0.056 |  |  |  |
|  |  |  | MAF | 0.263 | 0.31 | 0.15 | 0.08 |
| ***rs6927072*** | Intron | T>G | aa | 0.424 |  |  |  |
|  |  |  | Aa | 0.428 |  |  |  |
|  |  |  | AA | 0.148 |  |  |  |
|  |  |  | MAF | 0.359 | 0.28 | 0.20 | 0.21 |
| ***rs2982683*** | Intron | C>T | aa | 0.508 |  |  |  |
|  |  |  | Aa | 0.361 |  |  |  |
|  |  |  | AA | 0.132 |  |  |  |
|  |  |  | MAF | 0.312 | 0.31 | 0.20 | 0.00 |
| ***rs3020434*** | Intron | C>T | aa | 0.617 |  |  |  |
|  |  |  | Aa | 0.35 |  |  |  |
|  |  |  | AA | 0.034 |  |  |  |
|  |  |  | MAF | 0.209 | 0.28 | 0.02 | 0.14 |
| ***rs9340835*** | Intron | G>A | aa | 0.511 |  |  |  |
|  |  |  | Aa | 0.417 |  |  |  |
|  |  |  | AA | 0.071 |  |  |  |
|  |  |  | MAF | 0.28 | 0.36 | 0.21 | 0.26 |
| ***rs12199722*** | Intron | A>G | aa | 0.068 |  |  |  |
|  |  |  | Aa | 0.417 |  |  |  |
|  |  |  | AA | 0.515 |  |  |  |
|  |  |  | MAF | 0.276 | 0.33 | 0.04 | 0.00 |
| ***rs926777*** | Intron | A<C | aa | 0.643 |  |  |  |
|  |  |  | Aa | 0.289 |  |  |  |
|  |  |  | AA | 0.068 |  |  |  |
|  |  |  | MAF | 0.212 | 0.22 | 0.50 | 0.45 |
| ***rs1709183*** | Intron | C<T | aa | 0.143 |  |  |  |
|  |  |  | Aa | 0.37 |  |  |  |
|  |  |  | AA | 0.487 |  |  |  |
|  |  |  | MAF | 0.327 | 0.27 | 0.46 | 0.35 |
| ***rs1884051*** | Intron | G<A | aa | 0.086 |  |  |  |
|  |  |  | Aa | 0.331 |  |  |  |
|  |  |  | AA | 0.583 |  |  |  |
|  |  |  | MAF | 0.252 | 0.30 | 0.45 | 0.42 |
| ***rs2982712*** | Intron | T<C | aa | 0.169 |  |  |  |
|  |  |  | Aa | 0.571 |  |  |  |
|  |  |  | AA | 0.259 |  |  |  |
|  |  |  | MAF | 0.455 | 0.47 | 0.23 | 0.00 |
| ***rs3020407*** | Intron | G>A | aa | 0.135 |  |  |  |
|  |  |  | Aa | 0.383 |  |  |  |
|  |  |  | AA | 0.481 |  |  |  |
|  |  |  | MAF | 0.327 | 0.27 | 0.40 | 0.08 |
| ***rs3798577*** | mrna-utr | T>C | aa | 0.331 |  |  |  |
|  |  |  | Aa | 0.469 |  |  |  |
|  |  |  | AA | 0.173 |  |  |  |
|  |  |  | MAF | 0.421 | 0.47 | 0.40 | 0.41 |
| ***rs4870056*** | Intron | A<G | aa | 0.395 |  |  |  |
|  |  |  | Aa | 0.44 |  |  |  |
|  |  |  | AA | 0.165 |  |  |  |
|  |  |  | MAF | 0.385 | 0.40 | 0.34 | 0.40 |
| ***rs7743290*** | Intron | T>G | aa | 0.045 |  |  |  |
|  |  |  | Aa | 0.424 |  |  |  |
|  |  |  | AA | 0.53 |  |  |  |
|  |  |  | MAF | 0.256 | 0.19 | 0.38 | 0.37 |
| ***rs9322335*** | Intron | T<C | aa | 0.53 |  |  |  |
|  |  |  | Aa | 0.346 |  |  |  |
|  |  |  | AA | 0.124 |  |  |  |
|  |  |  | MAF | 0.297 | Not recorded | 0.44 | 0.31 |
| ***rs9322336*** | Intron | C<T | aa | 0.03 |  |  |  |
|  |  |  | Aa | 0.372 |  |  |  |
|  |  |  | AA | 0.598 |  |  |  |
|  |  |  | MAF | 0.216 | 0.17 | 0.40 | 0.00 |
| ***rs1709181*** | Intron | ? | aa | 0.109 |  |  |  |
|  | Minor allele: A |  | Aa | 0.455 |  |  |  |
|  |  |  | AA | 0.436 |  |  |  |
| ***rs9322334*** | Intron | ? | MAF | 0.336 | Not recorded | -Not recorded | Not recorded |
|  | Minor allele: A |  | aa | 0.553 |  |  |  |
|  |  |  | Aa | 0.342 |  |  |  |
|  |  |  | AA | 0.105 |  |  |  |
|  |  |  | MAF | 0.276 | Not recorded | Not recorded | Not recorded |
