# Supplementary material for: Polymorphisms in the estrogen receptor alpha gene (ESR1), daily cycling estrogen and mammographic density phenotypes
Source: BMC Cancer. 2016 Oct 7;16:776. doi: 10.1186/s12885-016-2804-1 (PMC5055696; doi:10.1186/s12885-016-2804-1)
Supplement: Additional file 2: Table S2. — The linear association between the selected SNPs in the ESR1 region and absolute mammographic density. (DOC 47 kb) [file 12885_2016_2804_MOESM2_ESM.doc]

**Additional file 2: Table S2. The linear association between the selected SNPs in the ESR1 region and absolute mammographic density.**

| **SNPs** | **BMI variable** | **β-value** | **^c^95%CI** | ***P-value*** |
| --- | --- | --- | --- | --- |
|  |  |  |  |  |
| ***rs3020364*** | Ungrouped**^a^** | 7.04 | (-1.96, 16.0) | *0.124* |
|  | BMI mediansplit**^b^**  Low  High | 8.47  12.1 | (-2.64, 19.6)  (-5.97, 30.1) | *0.133*  *0.187* |
| ***rs2474148*** | Ungrouped**^a^** | 5.02 | (-3.88, 13.9) | *0.268* |
|  | BMI mediansplit**^b^**  Low  High | 4.54 | (-6.61, 15.7)  (-5.44, 30.3) | *0.421*  *0.170* |
| ***rs12154178*** | Ungrouped**^a^** | 2.49 | (-6.40, 11.4) | *0.581* |
|  | BMI mediansplit**^b^**  Low  High | 8.32  6.06 | (-2.24, 18.9)  (-11.8, 23.9) | *0.121*  *0.502* |
| ***rs2347867*** | Ungrouped**^a^** | 5.93 | (-2.55, 14.4) | *0.169* |
|  | BMI mediansplit**^b^**  Low  High | 11.9  6.15 | (2.33, 21.4)  (-12.0, 24.3) | *0.015*  *0.501* |
| ***rs6927072*** | Ungrouped**^a^** | 2.17 | (-6.26, 10.6) | *0.613* |
|  | BMI mediansplit**^b^**  Low  High | 9.11  3.21 | (-0.67, 18.9)  (-14.5, 20.9) | *0.068*  *0.719* |
| ***rs2982712*** | Ungrouped**^a^** | 8.38 | (-0.18, 16.9) | *0.055* |
|  | BMI mediansplit**^b^**  Low  High | 9.8  12.3 | (-0.55, 20.2)  (-5.02, 29.6) | *0.063*  *0.162* |
| ***rs3020407*** | Ungrouped**^a^** | 0.91 | (-7.54, 9.36) | *0.832* |
|  | BMI mediansplit**^b^**  Low  High | 3.6  6.65 | (-6.32, 13.5)  (-10.7, 24.0) | *0.474*  *0.449* |
| ***rs9322335*** | Ungrouped**^a^** | 10.1 | (0.91, 19.2) | *0.031* |
|  | BMI mediansplit**^b^**  Low  High | 13.4  10.8 | (2.51, 24.7)  (-8.04, 29.7) | *0.016*  *0.257* |

^a^Multivariable linear regression, adjusted by age, BMI, menarcheal age and parity.

^b^Multivariable linear regression, adjusted by age, menarcheal age and parity. BMI median split at kg/m^2^

^c^Confidence Interval.
